# GTR Treatment in Furcation Grade II Periodontal Defects with the Recently Reintroduced Guidor PLA Matrix Barrier: A Case Series with Chronological Step-by-Step Illustrations

**DOI:** 10.1155/2020/8856049

**Published:** 2020-12-16

**Authors:** Anton Friedmann, Andreas Stavropoulos, Hakan Bilhan

**Affiliations:** ^1^Chair and Head Department of Periodontology, School of Dentistry, Faculty of Health, University of Witten, Alfred-Herrhausen Str. 44 58455 Witten, Germany; ^2^Chair and Head Department of Periodontology, Faculty of Odontology, University of Malmö, Carl Gustafs väg 34 205 06 Malmö, Sweden; ^3^Division of Regenerative Dental Medicine and Periodontology, University Clinics of Dental Medicine, CUMD, University of Geneva, Geneva, Switzerland; ^4^Department of Periodontology, School of Dentistry, Faculty of Health, University of Witten, Alfred-Herrhausen Str. 45 58455 Witten, Germany

## Abstract

Molars with a furcation involvement (FI) exceeding grade 1 according to Hamp's classification are at approximately doubled risk of tooth loss. Guided tissue regeneration (GTR) is a regenerative approach in the treatment of periodontal defects and is aimed at achieving new clinical attachment formation. The aim of this case series was to assess the efficacy of a newly reintroduced polylactic acid (PLA) matrix barrier and to evaluate the feasibility of the surgical approach. 11 patients with an average age of 58.7 years were treated with GTR using a PLA matrix barrier. Patients were instructed not to brush and chew on the treated side for 4 weeks. A gentle clinical probing was performed after 6 months for the first time after surgery. The patients were included into individual maintenance program at three months' interval. The clinical improvement was expressed by reduced horizontal penetration of the probe accompanied by vast resolution of the vertical defect component. The change from grade II to grade I or complete resolution of the FI could be seen in 8 from 11 sites included. The newly reintroduced PLA matrix barrier showed promising results after a 12-month observation period with clinical attachment gain.

## 1. Introduction

Periodontal attachment loss in the furcation area plays a pivotal role in the long-term prognosis of molars in both the mandible and the maxilla. Thus, molars with a furcation involvement (FI) exceeding grade 1 according to Hamp's classification seem to be at vast risk of tooth loss within a 5-year observation period [1, 2]. The presence of FI was shown to approximately double the relative risk of tooth loss for molars maintained in supportive periodontal therapy for up to 10-15 years. The risk increased obviously sharply looking at the maintenance rates after 15 years, although the authors pointed out the study heterogeneity [3]. Prognosis of FI teeth should also consider the vertical subcategorization into subclasses A/B/C, which associates the case complexity with the infrabony extension of the periodontal pocket [4, 5]. The subclass C representing the vertical extension of FI into the apical 1/3 of the root length was shown to have the lowest ten-year survival rate in class II involved multirooted teeth. The subclasses A and B were associated with 91% and 67% survival rates over the same period, respectively [6]. The clinical long-term observations of the nonsurgical therapy followed by SPT lasting even for decades have been shown to leave the FI grade II without improvement and justify the surgical intervention [6]. Guided tissue regeneration (GTR) is a regenerative approach in the treatment of periodontal defects and is aimed at achieving new attachment formation in periodontally involved teeth [7, 8]. Several systematic reviews have shown greater probing depth reduction, clinical attachment gain, and gain in hard tissue with GTR compared with open flap debridement in both intrabony and FI grade II defects [9] [10]. In this context, despite the observation that a complete furcation closure may rarely occur, the evidence points to the fact that GTR may often convert grade II furcation defects to grade I, which improves the long-term tooth prognosis [11].

The GTR technique relies on the use of a physical barrier to prevent epithelial downgrowth on the exposed root surface which is known to hinder the formation of new attachment components [12]. During the regenerative processes, protease enzymes could harm, since tissues may not be completely mature in the beginning of healing [13]. The use of a physical barrier as a membrane helps the abovementioned matrix to mature undisturbed. Most of the resorbable periodontal membranes are subjected to proteolytic degradation over time, with the exception of the PLA barrier, which is degraded by hydrolysis instead of enzyme activity [14]. The PLA matrix barrier was reported to show positive results in previous case series published in the past [15–18]. The long-term stability achieved with the GTR technique using the PLA barrier has been reported for a period of 6 to 7 years previously [19].

In this case series, the authors report the outcomes in 11 consecutively treated patients by applying the Guidor matrix barrier using the MPPT protocol. The results obtained clinically and radiographically at 12 months are summarized.

## 2. Clinical Procedures

**Figure 1:**
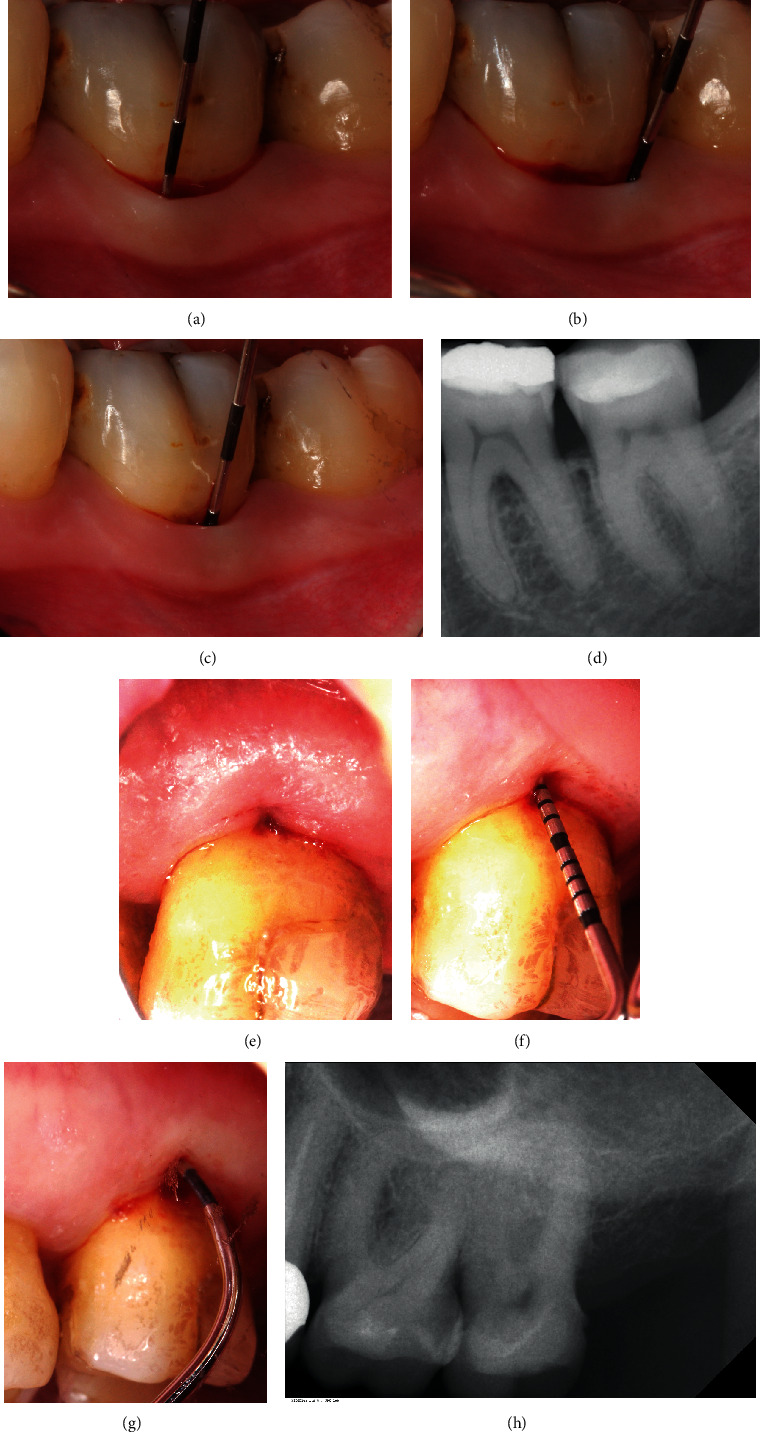
(a) Tooth 36 (FDI) baseline mesiobuccal VPD with 3 mm PPD F. (b) Tooth 36 (FDI) baseline distobuccal VPD with 3 mm PPD. (c) Tooth 36 (FDI) baseline 5 mm buccal VPD at the furcation entrance. (d) Tooth 36 (FDI) baseline periapical X-ray with furcation involvement grade 2. (e) Tooth 26 (FDI) baseline image of the gingiva margin at the furcation area. (f) Tooth 26 (FDI) baseline buccal 6 mm VPD at the furcation entrance. (g) Tooth 26 (FDI) baseline 6 mm HPD indicating buccal FI grade 2. (h) Tooth 26 (FDI) baseline periapical X-ray with furcation involvement grade 2.

All 11 patients were selected from the pool of SPT patients of the department of periodontology at Witten/Herdecke University, Germany, and Malmö University, Sweden. Each patient had to have a comprehensive SRP treatment in the past followed by several SPT visits documenting general improvement of periodontal conditions by reduced FMPS and FMBS levels and decreased periodontal probing depths. All patients assigned to the GTR therapy were non- or ex-smokers. The average age of the patients, 8 women and 3 men, was 58.7 years. Mandibular molars showing persistent FI grade 2 on the buccal or lingual aspect (Figures 1(a)–1(d)) and maxillary molars with a buccal FI grade 2 (Figures 1(e)–1(h)) and representing either subclass A or B received GTR treatment using a Guidor® matrix barrier (GUIDOR® Matrix Barrier-MSL (Molar Straight Large), Sunstar GmbH, Germany). This polymer is manufactured out of polylactides blended with a citric acid ester—compounds that have a history of more than 20 years of use in the food and medical industry.

**Figure 2:**
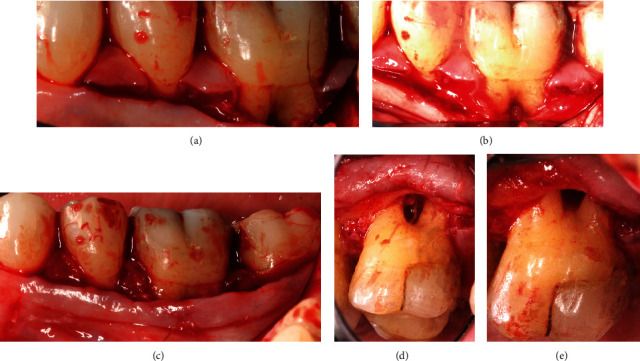
(a) The incision technique according to the modified papilla preservation method (MPTT) for accessing the buccal furcation in the mandibular molars (region 37-35). (b) Clinical image of the furcation defect after the preparation of the buccal full-thickness flap at tooth 36 which leaves the interproximal papillae in place. (c) The soft tissue preparation with deepithelized papillae prior to barrier insertion in the mandible. (d) Clinical image of the furcation area at tooth 26 before debridement after buccal full-thickness flap preparation with preserved papillae. (e) Clinical perspective of the furcation defect of tooth 26 after degranulation.

**Figure 3:**
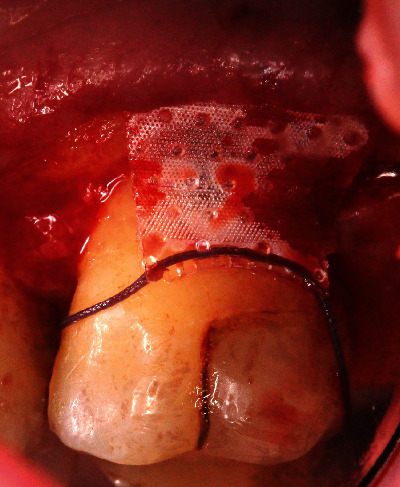
The Guidor matrix barrier in situ at the furcation entrance of tooth 26.

**Figure 4:**
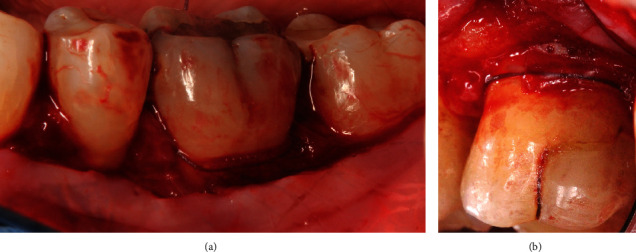
(a) The collar of the barrier carefully adapted to the root trunk slinging the integrated suture around the tooth placed subgingivally at tooth 36. (b) The collar of the barrier carefully adapted to the root trunk slinging the integrated suture around the tooth placed subgingivally at tooth 26.

**Figure 5:**
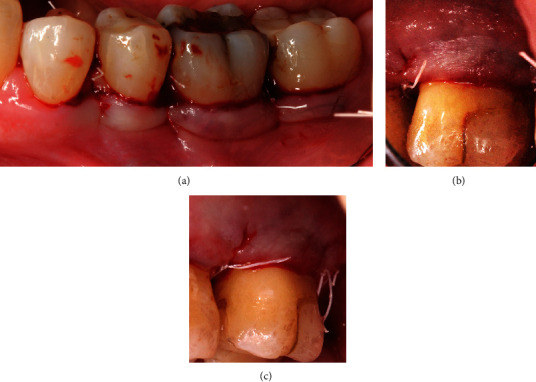
(a) The complete tensionless flap closure achieved by the coronally advanced flap (CAF) technique and the modified vertical mattress suture using PTFE 4.0 suture at tooth 36. The buccal aspect shows complete cover of the papillae and the barrier by the soft tissue. (b) The complete tensionless flap closure achieved by the CAF technique and the modified vertical mattress suture using PTFE 4.0 suture at tooth 26. The buccal aspect shows complete cover of the papillae and the barrier by the soft tissue. (c) The mesial view at the coronally repositioned flap at tooth 26.

All 11 cases were selected and treated by two periodontists (A.F. and A.S.), both calibrated regarding the surgical approach and GTR barrier. All surgeries were carried out under local anesthesia. The surgical approach was standardized. The Modified Papillae Preservation Technique (MPPT) was applied for incision and flap design (Figures 2(a)–2(c)) [20]. The papillae mesial and distal to the furcation treated were left in place without reflecting them. In the case of the distal molar, the sulcular incision was distally continued within the keratinized gingiva midcrestally in the edentulous zone. Vertical releasing incisions were not made. The buccal flap was reflected and released by a periosteal incision for coronal advancement before positioning the barrier. The root surfaces were thoroughly instrumented using Gracey curettes (Deppeler®, Deppeler SA, Rolle, Switzerland) and/or ultrasonic instruments (EMS, Munich, Germany), and the defects (Figure 2(d)) were completely degranulated (Figure 2(e)). All but two defects were nongrafted to allow for blood clot formation and maturation inside the furcation defect. The very first two cases were grafted by either autogenous bone chips or by CopiOs (Zimmer Biomet Deutschland GmbH, Freiburg i. Breisgau, Germany). The barrier (Figure 3) was shaped into the size overextending the defect margins by 2-3 mm. The collar of the barrier was carefully adapted to the root trunk slinging the integrated suture around the tooth subgingivally (Figures 4(a) and 4(b)) and placing the knot to the opposite side of the tooth. The papillae were deepithelized using scalpel blade and scissors. The coronal advancement of the flap by releasing the periosteum ensured complete cover of the membrane barrier and tensionless suture using the modified vertical mattress technique with 4.0 PTFE monofilament suture (Biotex®, Regedent, Dettelbach, Germany) [15, 17] (Figures 5(a)–5(c)). Moreover, the advanced flap margins were adjusted to completely cover the deepithelized papillae in a total incision extension.

**Figure 6:**
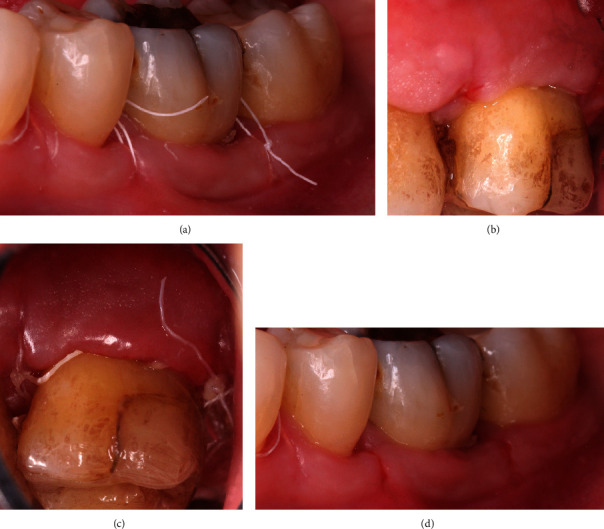
(a) Clinical image of tooth 36 at 2 weeks' visit indicates a minimal recession onset at the distal aspect before suture removal. (b) Clinical situation at tooth 36 after suture removal at the same visit. (c) Clinical situation 2 weeks post-op at tooth 26 shows complete cover of the barrier without any change in the level of the gingival margin before suture removal. (d) Dame visit, clinical situation after suture removal at tooth 26, mesial view.

**Figure 7:**
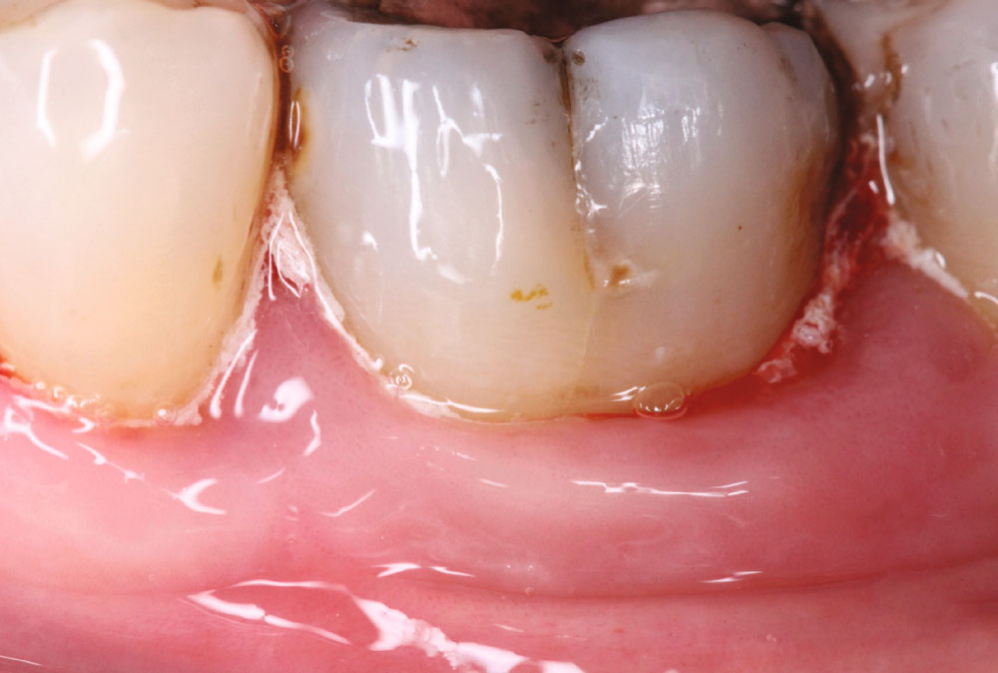
Four weeks' post-op image of tooth 36, taken following a professional tooth cleaning session.

**Figure 8:**
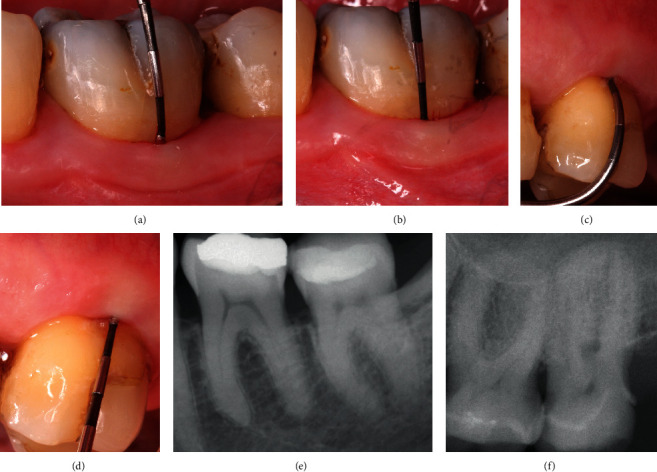
(a) Months' observation at tooth 36 reveals a 2 mm buccal HPD indicating the relevant improvement in the furcation area. (b) 12-month buccal VPD at 36 equals 3 mm depth, displaying a valuable improvement in vertical dimension. (c) 18-month observation displays a 3 mm of horizontal penetration depth (HPD) at tooth 26, indicating conversion from FI grade 2 to grade 1. (d) 18-month observation displays a 3 mm of vertical penetration depth (VPD) at tooth 26n from the buccal, indicating clinically relevant improvement in this dimension. (e) Periapical radiograph of tooth 36 after 12 months confirms clinical assessments and corroborates the improvement in the furcation area. (f) Periapical radiograph of tooth 26 after 30 months corroborates clinical measurements and confirms the success of the treatment.

The post-op regimen included patient's instruction to abstain from mechanical plaque control in the treated area for several weeks and to use Chlorhexidine (Chlorhexamed GlaxoSmithKline Consumer Healthcare GmbH & Co. KG, Munich Germany) mouth rinse twice a day instead. Doxycycline (200 mg/day) for duration of 10 days and analgesic medication (ibuprophen 600 mg/3x daily) on demand were administered; patients were rescheduled for weekly control visits. Sutures (Figures 6(a) and 6(c)) were removed after 14 days (Figures 6(b) and 6(d)), and the mouth rinse was thereafter substituted by the local use of Chlorhexidine gel (Chlorhexamed GlaxoSmithKline Consumer Healthcare GmbH & Co. KG, Munich, Germany) in the wound area. Patients were instructed not to brush and chew on the treated side for another 4 weeks (Figure 7). Clinical images were taken at every control visit, and the X-ray was repeated at the end of the observation period after 12 and 18 months (Figure 8(e)) and 30 months, respectively (Figure 8(f)). The gentle clinical probing was performed after 6 months for the first time after surgery. Nevertheless, the patients were included into individual maintenance program at three months' interval.

## 3. Results and Discussion

The clinical improvement was expressed by reduced horizontal penetration of the probe (Figures 8(a) and 8(c)) accompanied by vast resolution of the vertical defect component (Table 1) (Figures 8(b) and 8(d)), which also often could be followed radiographically (Figures 8(e) and 8(f)). The change from grade II to grade I or complete resolution of the FI was assessed in 9 from 11 sites included (Table 1). Improvement of clinical outcomes for buccal grade II furcation defects by treatment with GTR and class II to class I furcation conversion is an utmost anticipated success criterion for more than 20 years [21–23]. The complete furcation closure had been achieved in 50% of molars with extensive bone loss [24].

Although all FI defects were assigned to the subgroups A and B, the improvement in vertical attachment level was considerable in all sites. Three teeth with initial FI grade II displayed almost unchanged horizontal attachment levels at the final examination visit 12 months after GTR surgery. Both of these nonresponding sites were characterized by unfavorable soft tissue position associated with a deep recession and an almost opened furcation fornix; all three teeth showed also a wide divergence angle of the roots. Local factors like these are known to be negative predictors for the regenerative outcome [24, 25].

The successful clinical closure of grade II furcations at 1 year following combination therapy with an ePTFE membrane and DFDBA had been shown [26, 27]. However, according to the properties of the barrier material, just two initially enrolled cases were grafted by autogenous bone chips or a bone substitute. Thereafter, the grafting of the furcation area was omitted.

The reviews of histological outcomes in GTR procedures published a decade ago as a recent one both demonstrated favorable histologic healing after the use of a barrier membrane along with a grafting material and being superior to the results after open flap debridement [28, 29]. The long-term observations confirm the stability of newly gained clinical attachment level over decades, once the treatment achieved sufficient attachment gain evaluated within first 6 to 12 months post-op [30].

The clinical effect in treating the degree II furcation with GTR including or excluding the bone grafting appears debatable. Studies which were looking at additional effect of a graft missed to show the level of statistically significant difference between the two options, indicating thereby that the additional effect for the combined treatment in intrabony and in furcation defects was underestimated [11, 31]. Considering the mechanical properties of the PLA barrier such as stiffness and plasticity, both responsible for a valuable space maintaining capacity, no substitute material was used in most cases in the present series. Nevertheless, the GTR treatment regimen was successful in terms of clinical attachment gain in 8 of 11 furcation defects.

On the other hand, the space maintaining capacity of titanium-reinforced expanded polytetrafluoroethylene membranes was helpful even in reducing the negative impact of an unfavorable defect morphology as shown in a controlled clinical trial [32]. The PLA barrier, however, being biodegradable without a need of a reentry for membrane removal offers an obvious advantage over PTFE membranes. The integrated and degradable suture for fixing the barrier collar at the tooth neck appears an appropriate prerequisite for successful adaptation and immobilization of the barrier over the extension of the bony defect.

Numerous studies reported the MPPT as applied in all 10 cases effective in support of new attachment formation in infrabony and furcation defects [20, 33]. The recession of gingival margin was estimated to extend for 1 mm more compared to the baseline assessment. This tendency was in agreement with the data published from several multicenter studies on GTR in infrabony and furcation defects [34, 35].

Several factors at the patient level as at tooth level may counteract with the healing and thereby impair the long-term outcome. Patient's lifestyle-related factors such as smoking, plaque control, and compliance with maintenance procedures are to consider as well as wound stability and disclosure of the barrier infection by periodontal pathogens from the oral cavity [36]. The initial healing was uneventful in all 11 patients resulting in primary wound closure and wound stability during the first weeks of post-op monitoring. Patient's compliance may retrospectively be accounted as high. None of them reported late complications in the treated area. The patient-related perception of the applied surgical method and the material used were in complete agreement with previously reported outcomes [37].

According to improvement of clinical attachment level with and without the use of bone substitute in 8 of 11 cases, the membrane stabilization may be considered as one of the key factors for successful regeneration. The utilized matrix barrier here with embedded suture and high level of plasticity despite certain rigidity gives the clinician the possibility to easier adapt the barrier upon the defect and stabilize it even neglecting the physical support by a bone substitute.

The results obtained clinically and radiographically at 12 and 18 months indicate the potential of the matrix barrier and the constraints of its sole use under complex conditions for achieving new clinical attachment in the furcation areas. However, the recent systematic review and the meta-analysis of surgical treatment options in FI multirooted teeth revealed superior outcome for the regenerative strategies in general when compared to conventional flap surgery [38]. Hence, the long-term stability of the results will depend on the patients' compliance. It is known that the clinical improvements after regenerative treatment can be preserved on a long-term basis on the majority of treated sites, provided that patients do not smoke, keep high oral hygiene standards, and regularly attend the SPT.

## 4. Conclusions

This case series confirms that sound clinical improvements can be in general achieved with the use of the Guidor matrix barrier as a regenerative treatment in furcation grade II defects. Further, the unsatisfactory results obtained in 2 cases herein also point to the limits of the procedure in complex situations with deficient amount of soft tissue and unfavorable root morphology.

## Figures and Tables

**Table 1:** Data showing patient details and outcome of the treatment.

Pat.	Age	Tooth	Baseline					Surgery	Graft +/−	12 months					*Δ* (BL–12 mo)	
Pat.+gender	Born	(FDI+site)	PPD	REC	VAL	HAL	FI grade	Year	Type or 0	PPD	REC	VAL	HAL	FI grade	VAL	HAL
1/f	1966	47 b	6	5	11	6	II	April 2016	Autogenous	3	4	7	3	I	+4	+3
2/m	1954	37 b	5	1	6	6	II	Nov. 2016	CopiOs	3	2	5	3	I	+1	+3
3/f	1963	47 b	5	0	5	5	II	Jan. 2017	0	2	1	3	1	0	+2	+4
4/f	1970	37 b	4	1	5	4	II	Aug. 2017	0	2	1	3	2	I	+2	+2
5/f	1956	36 b	5	2	7	6	II	Sep. 2017	0	2	1	3	2	I	+4	+4
6/f	1950	46 ling	6	0	6	6	II	Sep. 2017	0	4	0	4	6	II	+2	0
7/m	1957	46 b	6	3	9	8	II	Dec. 2017	0	3	5	8	6	II	+1	0
8/m	1957	26 b	6	4	10	8	II	Dec. 2017	0	4	4	8	4	II	+2	+4
9/m	1955	46 ling	6	2	8	6	II	Jan. 2018	0	2	2	4	3	I	+4	+3
10/m	1955	26 b	6	2	8	8	II	Jan. 2018	0	3	2	5	3	I	+3	+5
11/f	1976	36	6	0	6	6	II	2017	0	3	0	3	3	I	3	3
∅															+2.6	+2.8

## Data Availability

Data is available on request through the first author Prof. Dr. A. Friedmann. However, it should be noted that in Germany details about patients are restricted due to the data protection laws.

## Abbreviations

GTR: Guided tissue regeneration
FI: Furcation involvement
SPT: Supporting periodontal treatment
SRP: Scaling and root planing
MPPT: Modified Papillae Preservation Technique
PLA: Polylactic acid
CAL: Clinical attachment level
CHX: Chlorhexidine
ePTFE: Expanded polytetrafluoroethylene
DFDBA: Demineralized freeze-dried bone allograft
FMPS: Full mouth plaque score
FMBS: Full mouth bleeding score.
